# Rebuildable Silver Nanoparticles Employed as Seeds for Synthesis of Pure Silver Nanopillars with Hexagonal Cross-Sections under Room Temperature

**DOI:** 10.3390/nano13071263

**Published:** 2023-04-03

**Authors:** Pengfei Yang, Yu Liang, Daxiao Zhang, Shaobo Ge, Shijie Li, Xichao Liang, Jin Zhang, Yingxue Xi, Yan Zhang, Weiguo Liu

**Affiliations:** 1 Shaanxi Province Key Laboratory of Thin Films Technology and Optical Test, Xi’an Technological University, Xi’an 710032, China; 2 State Key Laboratory of Precision Spectroscopy, East China Normal University, Shanghai 200062, China; 3 School of Physics and Technology, Wuhan University, Wuhan 430072, China; 4 Research and Application of Regenerative Cellulose Fiber Key Laboratory of Sichuan Province, YiBin Grace Group Co., Ltd., Yibin 644000, China

**Keywords:** silver nanoparticle seed, silver nanopillar synthesis, high repeatability, room temperature

## Abstract

Silver nanopillars with strong plasmonic effects are used for localized electromagnetic field enhancement and regulation and have wide potential applications in sensing, bioimaging, and surface-enhanced spectroscopy. Normally, the controlled synthesis of silver nanopillars is mainly achieved using heterometallic nanoparticles, including Au nanobipyramids and Pd decahedra, as seeds for inducing nanostructure growth. However, the seed materials are usually doped in silver nanopillar products. Herein, the synthesis of pure silver nanopillars with hexagonal cross-sections is achieved by employing rebuildable silver nanoparticles as seeds. An environmentally friendly, stable, and reproducible synthetic route for obtaining silver nanopillars is proposed using sodium dodecyl sulfate as the surface stabilizer. Furthermore, the seed particles induce the formation of regular structures at different temperatures, and, specifically, room temperature is beneficial for the growth of nanopillars. The availability of silver nanoparticle seeds using sodium alginate as a carrier at different temperatures was verified. A reproducible method was developed to synthesize pure silver nanopillars from silver nanoparticles at room temperature, which can provide a strategy for designing plasmonic nanostructures for chemical and biological applications.

## 1. Introduction

Owing to the favorable plasmon properties [1,2,3,4,5], silver nanopillars (SNPs) with anisotropic growth have been applied extensively in nanoscale optical devices [6,7,8], such as the nanowires used for plasmon waveguiding, which can realize light guiding with field confinement beyond the diffraction limit, providing fundamental building blocks for nanophotonic integrated circuits [9]. A silver nanorod can be used as an optical antenna for selectively extracting a near-field component, such that the light output from the silver nanorod mimics the emission of a plasmonic nanolaser [10]. A single silver nanorod used as a Fabry–Perot nanocavity provides a novel route for the manipulation of excitons in semiconductors [11]. As a result, the preparation of asymmetrical silver nanostructures has garnered increasing attention in recent years [12,13,14,15,16,17,18]. The most convenient approaches to synthesizing these silver nanostructures with specific sizes involve chemical reduction [19,20,21], such as the solvothermal method with a polyol, the seed directing method, or light-induced methods, among others [22,23,24,25,26,27,28,29,30]. In particular, seed particles and temperature play key roles in the synthesis of SNPs because they govern the shapes of the resulting nanostructures [31,32,33,34,35,36,37].

Temperature dominates the growth of silver nanostructures, especially with the solvothermal method [38]. Mahmoud and co-workers found that a rapid stirring rate disrupted symmetrical growth to favor nanopillar formation [39]. Meanwhile, a polyol serves as the solvent and reductant, and since this process is highly temperature-dependent, this leads to lower repeatability [40,41]. Therefore, a mild reaction could improve the controllability of nanostructures’ morphologies, and nanoseeds induce directional growth. Some foreign materials, such as gold or palladium, are typically employed as seeds for preparing SNPs [42,43]. These metal particles are prepared using some convenient and reproducible methods. However, silver nanoparticles are the optimal seed material due to their optical properties being similar to those of SNPs, which make SNPs exhibit consistent optical properties. Unfortunately, there are no controllable methods for preparing rebuildable silver nanoparticles that can serve as silver crystal seeds, and, therefore, such efforts often generate more unexpected results [44]. The present work describes the preparation of unique silver nanoparticles without a surface stabilizer. The developed approach relies on sodium alginate, and the products maintain high stability in deionized water, thus enabling the subsequent rebuilding of silver nanostructures with different shapes [45,46].

Considering that the optimal carrier for silver nanoparticles is a natural polymer, this study employed sodium dodecyl sulfate as a surface stabilizer for synthesizing silver nanostructures, owing to its favorable interactions with polymers [47,48,49,50,51]. This method was used to obtain asymmetrical SNPs with hexagonal cross-sections at room temperature in high yield with high repeatability. The growth mechanism of the SNPs was also elucidated via the characterization of the nanostructures. The rebuildable silver nanoseeds played a key role in inducing directional accumulation. Furthermore, the effects of various amounts of silver nanoparticles on the synthesis of silver nanostructures were evaluated. Temperature was demonstrated to be an important factor during the preparation, and the distinct products obtained at different reaction temperatures are discussed.

## 2. Materials and Methods

### 2.1. Materials

Sodium alginate (SA; 98%) was purchased from Shanghai Yuanye Bio-Technology Co., Ltd. Sodium dodecyl sulfate (SDS) and AgNO_3_ (99.8%) were purchased from Sinopharm Chemical Reagent Co., Ltd. (Shanghai, China). L-Ascorbic acid (AA; 99%) and sodium borohydride (NaBH_4_; 99%) were purchased from Sigma-Aldrich. Deionized water with a resistivity of 18.2 MΩ cm was obtained from a Direct-Q 5 UV water purification system and was used throughout all the experiments.

### 2.2. Methods

#### 2.2.1. Preparation of Silver Nanoparticles

Silver nanoparticles were synthesized according to the protocols described by Liang et al. [52,53]: by directly reducing Ag^+^ ions in sodium alginate (SA) solutions containing NaBH_4_ as the reducing agent. Specifically, 0.05 g of SA powder was dissolved in 500 g of deionized water to prepare a 0.01 wt. % SA solution. Subsequently, a AgNO_3_ solution (100 mL; 10 mM) was added to the SA solution under magnetic stirring. After 30 min of stirring, 50 mL of a 0.1 M NaBH_4_ solution was added, and the mixture was stirred for 2 h. The resulting solution was then dialyzed with regenerated cellulose tubes (*M_w_* cutoff, 8000) against deionized water containing NaBH_4_ for 3 days.

#### 2.2.2. Silver Nanopillar Synthesis

A 5 mM SDS solution was prepared in advance by dissolving 144.2 mg of SDS powder in 100 mL of deionized water and stirring for 30 min to ensure complete dissolution. Then, 15 mL of the SDS solution (5 mM) was stirred at 27 °C to establish a homogeneous reaction environment. Next, AgNO_3_ (0.01 M; 400 μL), AA (0.1 M; 50 μL), and 4 μL of the silver nanoparticle suspension were added to the SDS solution rapidly in sequence, and the final mixture was stirred continuously for 1–3 h. After the reaction was complete, 2 centrifugations (5000 rpm, 10 min each) were carried out within 30 min, and the precipitates were isolated, rinsed with deionized water, and then stored in an aqueous solution. For further characterization, natural air drying resulted in a certain amount of silver nanopillars. An SA solution (5 × 10^−4^ mg/mL; 50 μL) without nanoparticles was used instead of the silver nanoparticle suspension in blank experiments.

### 2.3. Characterization

Scanning electron microscopy (SEM) images of the prepared samples were obtained using a Hitachi S-4800 (Tokyo, Japan) with an accelerating voltage of 5 kV. Transmission electron microscopy (TEM) images, high-resolution transmission electron microscopy (HRTEM) images, energy-dispersive spectra (EDS), and selected area electron diffraction (SAED) patterns of the silver nanopillars were obtained using a Tecnai G2 F20 (FEI Ltd., Hillsboro, WA, USA) and a JEM-2010 FEF (JEOL Ltd., Tokyo, Japan) with an accelerating voltage of 200 kV. The X-ray diffraction (XRD) patterns of the dried nanostructures were recorded using a SmartLab X-ray diffractometer (Rigaku, Tokyo, Japan, Cu Kα radiation) in the 2θ range of 30°–90°.

## 3. Results

### 3.1. Pure Silver Nanopillars with Hexagonal Cross-Sections

**Figure 1:**
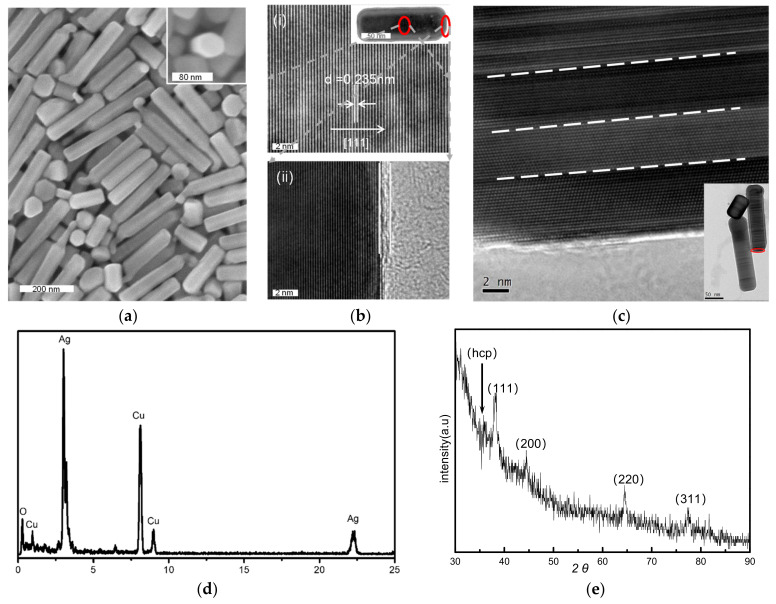
(**a**) The scanning electron microscopy (SEM) image of silver nanopillars (SNPs) synthesized from silver nanoparticles in 1 h, with candle-like structures and other by-products; inset shows the hexagonal cross-section of a nanopillar. (**b**) (**i**) The high-resolution transmission electron microscopy (HRTEM) image showing the lattice fringes of the SNP in the inset; (**ii**) The HRTEM image of the amorphous epitaxial layer on the side of SNP in the inset. (**c**) The HRTEM image of the nanopillar in the inset, revealing the lattice fringes and dislocations between layers at the one nanopillar’s side. (**d**) The energy-dispersive spectrum (EDS) spectrum of SNPs. (**e**) The X-ray diffraction (XRD) pattern of SNPs containing four peaks consistent with face-centered cubic (fcc) silver crystal structures.

The SNPs were synthesized in an hour by using silver nanoparticles as seeds, as shown in Figure 1a, and the hexagonal cross-sections can be observed in the inserted image. The results include nanopillars with clear edges along the surfaces and ends, as well as candle-like nanopillars with ‘wicks’, and by-products with other shapes. Some nanoplates were hexagonal, similar to the cross-sections of the silver nanopillars. The inset in Figure 1b(i) presents the morphology of a silver nanopillar, and Figure 1b was obtained using high-resolution transmission electron microscopy (HRTEM). The lattice spacing of 0.235 nm in Figure 1b(i) corresponds to the {111} of silver, which indicates that the silver nanopillars grew in the <111> direction. The amorphous epitaxial layer (AEL) at the edge in Figure 1b(ii) corresponds to the surface stabilizers surrounding the SNPs. The dislocation between layers on one side of a nanopillar is shown in Figure 1c. The energy-dispersive spectrum (EDS) analysis of the SNPs revealed peaks associated with silver and Cu, as shown in Figure 1d, wherein the Cu peaks were derived from the copper grid. Besides the typical 4 characteristic diffraction peaks of the X-ray diffraction (XRD) peaks in Figure 1e that correspond to the (111), (200), (220), and (311) planes of face-centered cubic (fcc) silver crystals, a peak also appears at ~35.9°, which can be indexed to 4H Ag [54].

### 3.2. Mechanism of SNPs’ Growth

**Figure 2:**
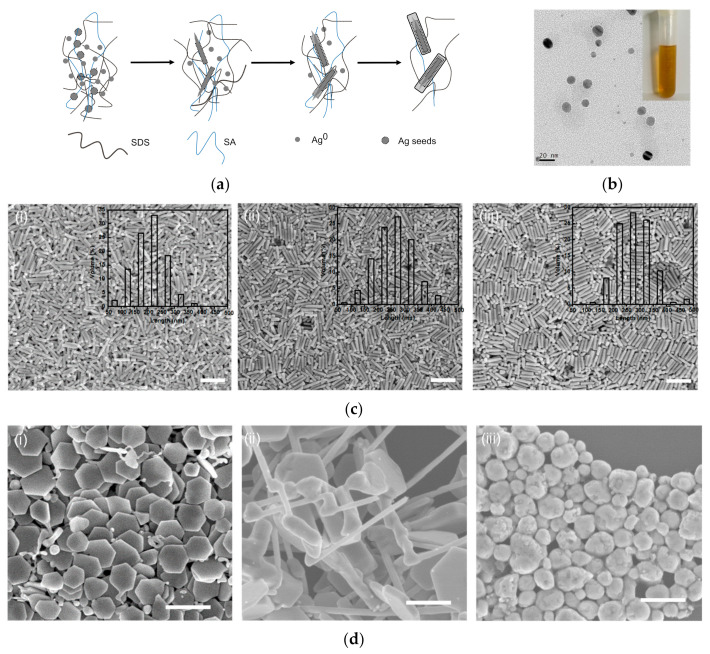
(**a**) Schematic depicting SNPs’ growth from silver nanoparticle seeds. (**b**) The transmission electron microscopy (TEM) image of silver nanoparticles; inset shows silver nanoparticle suspension. (**c**) SEM images and corresponding histograms (as shown in insets) showing the SNPs’ size distributions obtained during synthesis for (**i**) 1 h; (**ii**) 2 h; and (**iii**) 3.5 h. The scale bar is 500 nm. (**d**) The samples of the blank experiments using SA without nanoparticles and reacting for 2 h at (**i**) 0 °C; (**ii**) 27 °C; and (**iii**) 80 °C. The scale bar is 500 nm.

The transition from candle-like pillars to SNPs is illustrated in Figure 2a, wherein the two ends of the nanopillars tend to be flat. Since the silver nanoparticles are carried by polymer chains and their diameters are about 10 nm (Figure 2b), each nanoparticle can serve as a seed to rebuild silver nanostructures. Silver ions are reduced and initially adhere to the closest nanoparticle as more and more silver ions and atoms accumulate along the specific direction of <111> to form nanopillars and the ‘wicks’ of the candle-like nanopillars are covered in this process, that is, they grow layer by layer. This is a typical hcp (4H polytypic) phase involving the silver nanostructure growth process [55,56]. The silver atoms assemble along the long axis leading to the formation of a ‘wick’ at the initial stage of the reaction and then grow along the short axis for the formation of nanopillars.

The SNPs’ growth over time is shown in Figure 2c. There are many small by-products that are generated with the SNPs after growing for 1 h in Figure 2c(i), and most of the nanopillars are candle-like, although the lengths of these nanopillars have a wide distribution. The proportions of small particles and the candle-like nanopillars decrease after 2 h and 3.5 h, respectively, as shown in Figure 2c(ii,iii). Notably, the yield of uniform nanopillars after 3.5 h is higher than that after 1 h. The length distribution of the nanopillars in the inset of Figure 2c indicates that the sizes of the nanopillars range from 100~350 nm after 1 h and reach 200–350 nm after 3.5 h. That is, as the reaction proceeds, the length increase of the silver nanopillars gradually becomes slower. Importantly, the samples from the replicated experiments follow a similar tendency to the results in Figure 2c, thereby highlighting the high repeatability of the applied method, i.e., synthesizing SNPs by using silver nanoparticles as seeds. It is worth noting that the SNPs’ growth slows down as the reaction continues, owing to the reagent consumption. In the blank experiments, 50 μL of SA without nanoparticles was added at various temperatures, resulting in the formation of completely different shapes, as shown in Figure 2d. For example, the nanoplates with tails in Figure 2d(i) were obtained at 0 °C, the irregular bulk materials with microwires in Figure 2d(ii) were synthesized at 27 °C, and the defective egg-like morphology in Figure 2d(iii) was produced at 80 °C.

## 4. Discussion

### 4.1. Key Role of Silver Nanoparticles

**Figure 3:**
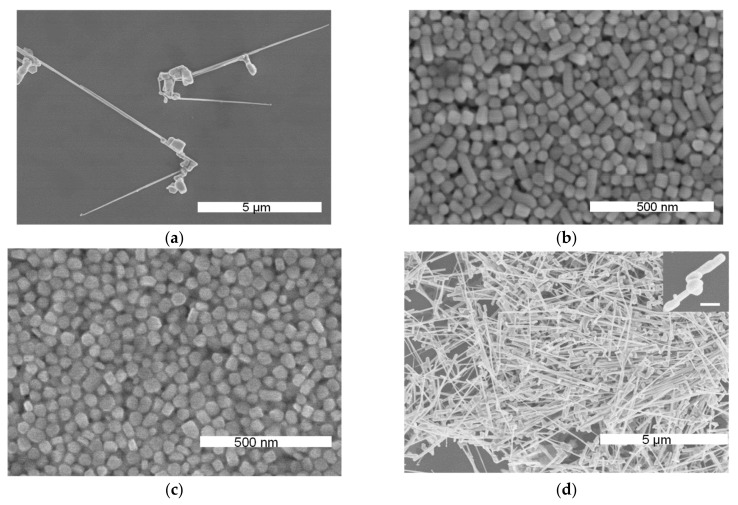
Images of the silver nanostructures prepared at 27 °C using different amounts of silver nanoparticles: (**a**) 0 μL; (**b**) 40 μL; (**c**) 200 μL; and (**d**) 400 μL, where the inset shows the irregular top, similar to nanopillars with nodes (the scale bar in inset corresponds to 200 nm).

Considering the key role of the silver nanoparticles in synthesizing nanopillars, different amounts of nanoparticles were used to prepare SNPs, and the results after 1 h (at the same temperature, 27 °C) are shown in Figure 3. The results of the experiment without silver nanoparticles in Figure 3a show microwires with irregular bulk on one side, which is similar to the microwires in Figure 2d(ii). Figure 3b,c shows images of the samples obtained using 40 μL and 200 μL of silver nanoparticles, respectively, which contain fewer nanopillars and more particles that are similar to the thick nanoplates. Notably, the particles in Figure 3c have rough surfaces and irregular edges. When the addition of nanoparticles was increased to 400 μL, as shown in Figure 3d, silver microneedles with abnormal tops on one end and sharp tips on the other end were produced. The inset in Figure 3d shows the nodes that were formed at the tops of these nanopillars.

Interestingly, microwires appear in the absence of nanoparticles or when there is an excessive number of nanoparticles in the reaction mixture. This is because there is excess polymer involved in the reaction. Without silver nanoparticles, the silver ions are reduced to form small particles in situ, and they then grow into microwires under the action of SDS, which acts as a kind of polymeric surface stabilizer. Introducing suitable proportions of the silver nanoparticles and SDS (i) allows the particles to aid in the nanostructure synthesis and (ii) enables the nanoparticle carrier—SA, to be involved in the reaction to induce SNP growth. As an increasing number of nanoparticles are added to the reaction, there are more seeds that can be used to rebuild nanostructures, and silver ions and atoms can quickly and simultaneously adhere to most of the nanoparticles. However, there are not enough silver ions to promote further growth during the reaction, and as a result, the sample in Figure 3b contains shorter structures than those in Figure 2a, and the sample in Figure 3c shows mostly plate-like bulk structures with irregular shapes. Meanwhile, thick nanoplates can ensure that nanopillars grow layer by layer. The SA content increases in the reaction when a large quantity of nanoparticles is added. The polymers of SA and SDS play crucial roles in the growth of silver structures, although the particles are used as seeds. This is consistent with the blank experiment without nanoparticles, in which there was an excess of the polymer in the reaction, and thus, microneedle structures are formed with abnormal nanopillars at one end.

### 4.2. Various Shapes at Different Temperatures

**Figure 4:**
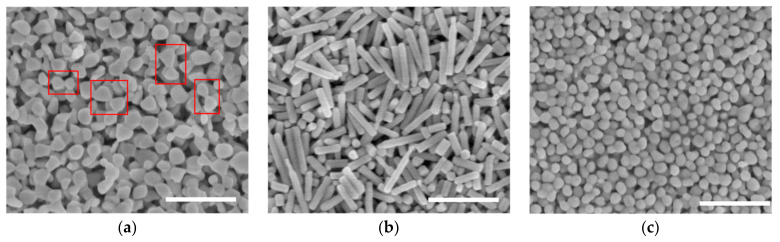
Images of the silver nanostructures prepared from 4 μL of silver nanoparticle suspension after 1 h at (**a**) 0 °C; (**b**) 27 °C; and (**c**) 80 °C. The scale bar is 500 nm.

To further examine the role that temperature plays in the synthesis of SNPs, 4 μL of a silver nanoparticle solution was employed in the reaction at different temperatures. The results after 2 h are shown in Figure 4. The irregular nanoparticles that are bonded with one another in Figure 4a were obtained at 0 °C, and some combinations adopted bowknot shapes. The discrete nanoparticles in Figure 4c were obtained at 80 °C, in stark contrast to the nanopillars in Figure 4b, which were synthesized at 27 °C. Considering the blank experiments (as shown in Figure 2d), it was determined that room temperature (27 °C) was optimal for growing nanostructures in a specific direction, such as nanopillars (Figure 4b) and bulk wires (Figure 2d(ii)). Furthermore, 80 °C could promote the isotropic growth of structures, such as the nanoparticles in Figure 4c and irregular particles with rough surfaces in Figure 2d(iii). Compared with the results obtained using silver seed particles, the carrier SA in the blank experiments at different temperatures tended to induce longer, twisted structures, such as the bulk wires in Figure 2d(ii), although the products included hexagonal nanoplates and nanoparticles at 0 °C and 80 °C, respectively. Moreover, the results of the blank experiments confirm that the seed nanoparticles induced the directional attachment of silver ions and atoms at corresponding temperatures, such as nanopillars at 27 °C and irregular spheres at 80 °C.

According to the various sample morphologies obtained using different temperatures and quantities of silver nanoparticles, we propose a potential mechanism for the formation of the SNPs. There is a mild reaction at room temperature when silver nanoparticles are added into the reaction mixture, and the silver ions and atoms aggregate in a specific direction using the seed particles as nuclei to form nanopillars with the longitudinal axes. Without silver seeds, the self-accumulation of the silver atoms and the growth of the nanostructures are dominated by the interactions involving SA and SDS, thereby resulting in long, irregular structures. The reaction at 0 °C generally remains steady, while the bonding between particles is induced by the SA and SDS polymers in the reaction, and, again, the particle formation is induced by silver seeds. Nanoplates with smooth surfaces and clear edges are obtained without silver nanoparticle seeds owing to the fine control of SA at freezing temperature, as demonstrated in our previous report [57]. This also explains why the nanostructures synthesized at room temperature in the blank experiment had smooth surfaces. Higher temperatures trigger a faster and more vigorous reaction at 80 °C. When seed particles are added to the reaction, the nanoparticles are rapidly encapsulated by the surrounding polymer, promoting the multidirectional growth of the silver nanoparticles to form a sphere-like morphology. The irregular particles observed in the blank experiment were produced by the SA and SDS, and the formation of rough surfaces was due to SA’s weaker control over the structures at relatively high temperatures.

## 5. Conclusions

Pure silver nanopillars with hexagonal cross-sections were synthesized by employing rebuildable silver nanoparticles as seeds. These seeds were carried by the natural polymer SA, using SDS as the surface stabilizer, thus providing an environmentally friendly, stable, and reproducible synthetic route for silver nanopillars. Various structures could be generated by controlling the reacting conditions, which indicates that the silver nanoparticles possess highly rebuildable properties. According to the product characterization and analysis of the experimental results, the relative proportions of the nanoparticles and SDS governed the production of nanopillars at 27 °C. Meanwhile, the seed particles could induce regular structures at different temperatures, although room temperature was beneficial for the growth of nanopillars. This report demonstrates a repeatable method for synthesizing silver nanopillars in a specific size range at room temperature. Furthermore, the nanoparticles generated at 80 °C verified the applicability of silver nanoparticle seeds when using SA as a carrier at different reaction temperatures.

## Data Availability

Data sharing not applicable. No new data were created or analyzed in this study. Data sharing is not applicable to this article.
